# Are Dutch patients willing to be seen by a physician assistant instead of a medical doctor?

**DOI:** 10.1186/1478-4491-10-28

**Published:** 2012-09-04

**Authors:** Luppo Kuilman, Roos MB Nieweg, Cees P van der Schans, Jaap H Strijbos, Roderick S Hooker

**Affiliations:** 1 Master Physician Assistant Program, School of Health Care Studies, Hanze University of Applied Sciences, Groningen, Groningen, The Netherlands; 2 Research and Innovation Group in Health Care and Nursing, Hanze University of Applied Sciences, Groningen, Groningen, the Netherlands; 3 Rehabilitation Medicine, University Hospital Groningen, Groningen, the Netherlands; 4 Nij Smellinghe Hospital, Drachten, the Netherlands; 5 The George Washington University, School of Public Health, Washington, DC, USA

**Keywords:** Physician assistants, Trade-off, Willingness

## Abstract

**Background:**

The employment of physician assistants (PAs) is a strategy to improve access to care. Since the new millennium, a handful of countries have turned to PAs as a means to bridge the growing gap between the supply and demand of medical services. However, little is known about this new workforce entity from the patient’s perspective. The objective of this study was to assess the willingness of Dutch patients to be treated by a PA or a medical doctor (MD) under various time constraints and semi-urgent medical scenarios.

**Methods:**

A total of 450 Dutch adults were recruited to act as surrogate patients. A convenience sample was drawn from patients in a medical office waiting room in a general hospital awaiting their appointments. Each participant was screened to be naive as to what a PA and a nurse practitioner are and then read a definition of a PA and an MD. One of three medical scenarios was assigned to the participants in a patterned 1-2-3 strategy. Patients were required to make a trade-off decision of being seen after 1 hour by a PA or after 4 hours by a doctor. This forced-choice method continued with the same patient two more times with 30 minutes and 4 hours and another one of 2 hours versus 4 hours for the PA and MD, respectively.

**Results:**

Surrogate patients chose the PA over the MD 96 % to 98 % of the time (depending on the scenario). No differences emerged when analysed by gender, age, or parenthood status.

**Conclusion:**

Willingness to be seen by a PA was tested a priori to determine whether surrogate Dutch patients would welcome this new health-care provider. The findings suggest that employing PAs, at least in concept, may be an acceptable strategy for improving access to care with this population.

## Background

At the turn of the century, the need for more medical doctors (MDs) in the Netherlands was predicted because an aging medical workforce and an aging population were constraining access [1]. One solution to this alignment problem was to redefine professional roles in health care and introduce a new professional: the physician assistant (PA) [2]. PA education in the Netherlands is a 30-month university program at a professional Master’s degree level. Graduates practice medicine in collaboration with and alongside MDs [3]. In the last decade, Australia, Canada, the Netherlands, and the United Kingdom have introduced PAs into their health-care system and the number of graduates is increasing steadily [4]. The health-care system of the United States introduced PAs in the late 1960s and in Canada in the 1990s [5]. How patients accept this new kind of health provider in these countries has been only partially explored.

Since the implementation of Dutch PA training in 2001, this workforce has grown from three in 2002 to over 650 by 2012. PAs work in a variety of medical specialties including general practice. There are approximately 65 000 registered physicians [6], serving a total population of 16.5 million. The PA cadre is small but expected to grow and supplement some portion of the medical service. While patient acceptance of Dutch PAs is only marginally known, some information exists outside the Netherlands. For example, a Scottish pilot study undertook patient acceptance interviews about the services they received from American PAs. One outcome was that all patients were willing to be seen by that PA again [7]. In another British study, most of the patients were satisfied with the PA’s care and only 7 % of the 167 interviewed patients opted to be seen by a doctor [8]. The perceptions of all 24 patients treated by the PAs in a Canadian orthopaedic surgical service were very positive [9]. These studies suggest that patients are generally accepting of services provided by PAs.

A report by the Dutch Council for Public Health and Healthcare (2002) suggested that acceptance of PAs should be a focus of research [10]. In 2008, the Health Council of the Netherlands reported observations of patients’ acceptance of non-physicians. The Health Council report indicated that patients are somewhat reticent towards non-physicians but are as accepting of the services of non-physicians as they are MDs [11]. Specific data regarding patients’ acceptance of PAs in the Netherlands has not been reported.

Since the 1970s, a few studies regarding patient acceptance of PAs have been undertaken. Nelson et al. [12] surveyed a total of 835 patients’ acceptance by these factors: latitude of delegated tasks, interpersonal manners and technical competence of PAs. Regarding technical competence, 89 % of patients reported their PAs being competent to very competent, and 86 % found their interpersonal manners to be professional. For routine technical procedures (e.g. injections, recording vital signs), 98 % of patients highly agreed that the tasks were delegable. Task acceptance such as first visit evaluations, house calls, and minor surgery ranged from 63 % to 75 % patient approval. However, 58 % of patients opposed to PAs providing prenatal care. Two-thirds (69 %) of the patients in the Nelson study agreed that the PA was able to take care of the patient if the doctor was out of town. Among all 449 respondents, only 20 % reported any negative attitude toward the PA.

PA acceptance appears to be influenced by many causes as outlined in an exploratory study undertaken in a rural American community. In a qualitative study, Baldwin et al. [13] found that community acceptance of PAs and nurse practitioners depended on satisfaction and willingness to seek care from either one. This was based on the perceptions and opinions of focus groups composed of four to nine community members. In general, it was concluded that community acceptance could be achieved if certain conditions were met. These conditions were personal factors that mainly embraced provider characteristics (e.g. friendliness and competence) whereas systemic factors covered conditions such as the need for collaboration between PAs and doctors, accessibility of care, and cost-containment. The systemic factors were mentioned as conditional acceptance and matched with the acceptance factors as studied by Nelson in 1974.

In an ethnographic evaluation of rural Texans [14], patient acceptance of single practice PAs was investigated using focus groups of citizens in small towns with a clinic; in all instances the townsfolk had positive feelings toward the PAs. The authors concluded that acceptance depends on the patients’ willingness to seek care by PAs and the feelings regarding the PA. One observation was that the PA and clinic reputation within the community had a predictive effect on acceptance.

Based upon these studies, we suggest that patient acceptance depends on multiple factors. Next to the conditional properties, such as personal and systemic factors, it appears that acceptance depends on the patients’ willingness to seek care when this new type of medical care provider delivers it.

Since the PA workforce in the Netherlands is early in development, we suggest that measurement of patient willingness towards PAs can be investigated as a precursor of patient acceptance, from a theoretical standpoint. An Australian-based study examined female willingness to choose care from PAs. In an outpatient clinic in Northern Queensland, women between 20 and 45 years old were screened for lack of knowledge about PAs. When presented with one of three scenarios, involving either a PA or a doctor with time choices, they chose care by the PA over 90 % of the time, regardless of decreasing time differences [15]. The findings of the Queensland study were limited in external validity due to the location and the restriction to women of childbearing age.

The primary objective of this European study is to investigate whether Dutch patients, recruited in a specialty care setting, are as willing to be treated by a PA instead of an MD. Because the Australian study lacked broader generalization, we included both genders in our study with a wider age base. Based upon this, a secondary outcome was if parenthood might influence the willingness of those patients where a scenario includes a mildly injured child. Finally, we have opted to also include male participants to see if patients’ willingness will reveal gender differences. By including both genders, our hypothesis is that the willingness of the patients to be seen by a PA will be significantly lower when compared with the Australian sample.

## Methods

We determined the sample size by applying following formula: $\left[DEFF*Np\left(1-p\right)\right]/\;[(d^{2}/{Z^{2}}_{1-\alpha /2}*\left(N-1\right)+p*\left(1-p\right)]\text{,}$ taking into account a population size **(N)** of 44.000 patients contacts, based upon the last annual amount of outpatient department consultations by cardiology, pulmonology and surgery. We set a confidence level of 95 % with an error margin of 5 % **(d)** and 1.0 for the design effect **(DEFF)** generally used for random samples. Given this fact and our hypothesized frequency $\left(p=50\%\;+/-\;5\right)$ of willingness among patients to participate in choosing between a PA and MD, a sample size of n = 381 was calculated to be appropriate. However, we expanded this survey to 450 subjects, using a convenience sample of 225 men and 225 women, to rule out the possibility of missing data and assure adequate representative data, thus enabling comparison of our female sample with the recruited sample in Australia.

### Design/population

Face-to-face interviews took place from November 2009 through April 2010. The questionnaire created a forced-choice strategy where participants were required to select either a PA or an MD for care, depending on the offered waiting times.

### Inclusion and exclusion criteria

Adults between the ages of 20 and 50 years were included. Individuals were eligible if they had not received services from a PA or a nurse practitioner. Those familiar with nurse practitioners were excluded because the providers may to some extent overlap in tasks, whereby a role-dependent bias could be introduced.

### Setting

Study candidates were recruited sequentially in the waiting rooms of their pulmonologist, cardiologist, or surgeon in the Nij Smellinghe hospital. This hospital contains 339 beds, covering all medical specialties, and is located in Drachten, a town with almost 45 000 citizens in the northern part of the Netherlands. Choosing a specialty care setting for recruitment was through agreement with the medical staff to participate in our study.

### Intervention

A research assistant approached each adult by opportunity, without predetermination, and requested his or her participation in this study. Each person was told the interview would take 15 minutes. Oral informed consent was obtained. The participants read the following role delineations of the PA and MD:

"Physician Assistant (PA): “*The PA is a clinician that has been clinically active in the Netherlands since the year 2000, and is a relatively new type of health professional in the medical domain. They conduct low to moderately complex medical tasks within a certain specialty. To become a PA, a professional Master’s degree course needs to be followed, which takes 2.5 years. Medical procedures performed by a PA are under the (in)direct supervision of a responsible medical specialist. Along with generating medical diagnoses, acquired via history taking and physical examination, PAs can also determine whether additional diagnostics (e.g. blood or urine samples or imaging such as X-ray) need to be requested. Based on the diagnosis, the PAs can decide which treatment is necessary. After that, they can execute medical technical interventions such as suturing, treating a sprained ankle, and assisting the specialist during surgery.*”"

"Medical Doctor (MD):*“A MD is a university trained professional with a 6-year basic training in medicine completed at a scientific Master’s level. !fter basic training, an MD may specialize, with further training ranging from at least 3 years and up to 6 years of further training. A MD is fully entitled to practice medicine under the law and therewith ultimately performs his/her own medical practice independently.*”"

Next all patients were confronted with one of the three scenarios, assigned to each in a 1-2-3 repeated sequence. The next three scenarios were used to determine whether *willingness* depended on the type of health problem:

"Scenario A:***“You stepped out of an automobile into a hole in the pavement and severely injured your ankle. The ankle is swollen and you are unable to put weight on your injured foot.***”"

"Scenario B:***“You have a 4 cm laceration on your forearm and blood was spurting before you could put a tourniquet on your arm.***”"

"Scenario C:***“Your 4-year-old daughter falls off the swing, hits her head on a rock, and has a 2 cm gash in the back of her head.***”"

All conversations and descriptions were in Dutch. No explanations were provided beyond the written material and the participants had to make all decisions based on what was presented to them on paper.

### Measures

After reading the assigned scenario, the patient selected to be treated by the PA or the MD with a waiting time of 1 hour versus 4 hours (timeslot 1). After the first choice was made, two sequential forced choices of 30 minutes versus 4 hours (timeslot 2) and 2 hours versus 4 hours (timeslot 3) were offered. The primary outcome was the willingness to be seen by a PA or an MD.

### Ethics

The Medical Ethics Committee of the University Medical Center Groningen concluded that this study would not be subject to the Medical Research Involving Human Subjects Act and could, therefore, be performed without further review.

### Statistical analysis

Demographic data were compiled by frequency and descriptive analysis. A one-sample binomial test – with the test proportion set at 0.5 – determined whether the proportion of PA versus MD, on the dichotomous variables, significantly differed from the hypothesized value. Cross-tabulation analysis assessment of the patients’ willingness rate, using a Pearson’s Chi-square test, was performed for the association between willingness, gender and parenthood. Statistical significance was determined a priori at the level of *P* < 0.05. All analyses were performed using the Statistical Package for the Social Sciences (release 17.0 SPSS Inc., Chicago, IL, USA).

## Results

The study included 225 men and 225 women. Male mean age was 39 years (SD 8 years) and female, 38 years (SD 8 years). The majority (66 %) of participants were follow-up patients; half (52 %) of the individuals visited the outpatient department for chronic disease management. Distribution was divided over the departments of pulmonology (159 patients), cardiology (155 patients), and surgery (136 patients). Approximately half of these patients had visited the outpatient department in the last year. Almost all (93 %) had received secondary or higher education and more than half (57 %) were married. Of the female participants, 165 had children compared with 146 of the men. The majority of all participants (85 %) were employed; 143 women and 26 men were employed part time (Table1).

**Table 1:** Demographics of investigated population

		**Frequency**	**Per cent**
**Age (mean, SD)**	male	39 (8.0)	.
	female	38 (8.0)	.
**Gender**	male	225	50.0
	female	225	50.0
**Type of patient**	new patient	153	34.0
	follow-up patient	297	66.0
**Inclusion per OPD**	pulmonology	159	35.3
	cardiology	155	34.4
	surgery	136	30.2
**More than one visits to OPD last year**	yes	256	56.9
	no	194	43.1
**Characteristic of health problem**	not sure	3	0.7
	acute	213	47.3
	chronic	234	52.0
**Marital status**	unanswered	1	0.2
	married	258	57.3
	not married	191	42.4
**Children**	unanswered	1	0.2
	yes	311	69.1
	no	138	30.7
**Highest level of education**	primary	30	6.7
	secondary	299	66.4
	tertiary	121	26.9
**Professionally occupied**	not working	71	15.8
	full-time	210	46.7
	part-time	169	37.6

OPD: outpatient department.

Patients’ willingness to opt for care delivered by PAs compared with that delivered by MDs, regardless of scenarios; almost all patients (96 % to 98 %; *P* <0.001) were unwilling to wait for a doctor’s treatment for 4 hours (see Tables 2 and 3). Of those few not selecting the PA, three women in the group of patients new to the clinic chose to wait for an MD; all other participants opted for the PA. In the follow-up patients, 12 selected an MD at timeslot 1, 9 at timeslot 2, and 14 for timeslot 3.

**Table 2:** Patients’ willingness to choose a physician assistant or a medical doctor

	**Category**	**N**	**Observed proportion**	**Test proportion**	**Asymptotic significance (two-tailed)**
**Timeslot 1**	PA	438	0.97	0.50	0.000^a^
	MD	12	0.03		
	Total	450	1.00		
**Timeslot 2**	PA	441	0.98	0.50	0.000^a^
	MD	9	0.02		
	Total	450	1.00		
**Timeslot 3**	PA	433	0.96	0.50	0.000^a^
	MD	17	0.04		
	Total	450	1.00		

^a^Based on Z approximation. MD: medical doctor; PA: physician assistant.

**Table 3:** Percentage of preference for physician assistant or medical doctor in the different scenarios and for the different timeslots

**Scenario**	**Timeslot 1**		**Total**	**Timeslot 2**		**Total**	**Timeslot 3**		**Total**
	**PA**	**MD**		**PA**	**MD**		**PA**	**MD**	
**A (n = 150)**	32.2	1.1	33.3	32.7	0.7	33.3	32.0	1.3	33.3
**B (n = 150)**	32.9	0.4	33.3	32.9	0.4	33.3	32.0	1.3	33.3
**C (n = 150)**	32.2	1.1	33.3	32.4	0.9	33.3	32.2	1.1	33.3

MD: medical doctor; PA: physician assistant.

Overall, 12 patients were willing to wait for an MD even though they had to wait 4 hours compared with seeing a PA within 1 hour. Concerning timeslot 2, nine patients chose to wait for an MD. Almost 4 % of the patients preferred the MD to the PA at timeslot 3. There was no significant association between the choices for a PA between the male (n = 25) and female (n = 13) participants at neither timeslot 1 (*P*: 0.079), timeslot 2 (*P* 0.312) nor timeslot 3 (*P* 0.458).

Willingness to wait for a PA in this cohort does not appear to be influenced by having children; only 9 who had children (*P* = 0.360) were willing to wait for an MD at timeslot 1; 7 (*P =* 0.560) at timeslot 2, and 12 (*P =* 0.808) at timeslot 3. The number of childless patients who preferred to wait for an MD in timeslot 1 was three (*P =* 0.99), timeslot 2 was two (*P =* 0.64), and timeslot 3 was five (*P =* 0.46).

Only six participants volunteered to justify their choices. They selected an MD for the following reasons:

“Seriously doubt the level of PAs and therewith in no way want to be helped by this type of provider”.

“Believe that by appointing more PAs, the level of the Dutch health care will decrease”.

“Fundamentally against being helped by lesser trained providers”.

“Prior negative experience with health care so and therefore not want to be treated by a trainee type of provider”.

“Not want to expose their child to a random type of provider, but the best care available”.

## Discussion

Contrary to our hypothesis that a male sample would show a decline in willingness, a high number of men chose to be seen by a PA instead of an MD. Since the convenience of time was the trade-off, this suggests the interviewees prefer the shorter wait to the familiarity with MDs. With minimal differences of affirmative responses among the three timeslots, the majority of the participants selected the PA. We suggest that patients, once informed about the competencies of lesser-trained professionals, may be prepared to take a certain risk as long as they gain quicker access to care. This effect could be due to the semi-dependent role of the PA [16]. One interpretation of the high patient acceptance rate is that the PA practices medicine under the supervision of a doctor; the patient may find this reassuring enough to influence their selection. Furthermore, participants were informed that PAs are capable of conducting low to moderately complex medical tasks. The high willingness rate of selecting the PA might reflect the non-life-threatening character of the described scenarios which are likely treatable by a PA. The findings of this study correlate with a similar undertaking in Vancouver, Canada that focused on mothers of children admitted to a pediatric emergency department. Doan and colleagues found that the willingness of surrogate patients and parents were also willing to accept the concept of a PA under similar scenarios described in this study [17]. Whether other types of first-aid providers might produce similar results opens opportunities for further research.

We offer some limitations to this work. In retrospect, the choice of a convenience sample is potentially a threat to the internal validity of the study. A randomized inclusion and assignment of the scenarios may have given the design a more sound character but there was no pre-selection in the sequence we used for participants and scenarios. Other limitations of this study are the fictional scenarios under which the participants made choices. Knowing they were not personally involved in the scenarios may have influenced the choice of an unknown type of provider over the doctor. The patients’ real-life distress, while waiting for, or having just met, their MD might also have influenced their choices. Another limitation is the design of the forced-choice strategy. The differences of the three timeslots could have been decreased and a slot in which patients would have been able to access the services of a PA or MD having a 30-minute difference in waiting time might have revealed different findings. Furthermore, a small group of patients in one section of one city in a doctor’s office does not represent the Dutch culture or nation as a whole.

We suggest that further research should include scenarios that describe a few severity levels in the range of low to moderately complex problems and upwards to life-threatening health scenarios. Acute exacerbations of chronic diseases might add some distinctions between the two providers.

The large number of patients who are preferentially selecting treatment by a PA should be viewed with caution. In this case, patient actors had an option to wait for an MD. The high number of patients willing to been seen by a PA instead of an MD stands in contrast to a 2002 study by Larkin and Hooker [18], where 79 % of the patients (n = 507) in an urgent care setting were only willing to be seen by an MD. This contrast may be due to the unsupervised status of the PA provider in their study. Some patients, who had reasons not to choose a PA, anecdotally expressed their concerns about the introduction of PAs. The chance to systematically explore such concerns was missed due to the lack of a mixed methods approach (quantitative versus qualitative) [19].

## Conclusions

Our study of Dutch surrogate patients, recruited in a specialty care setting, further identifies that a large majority of individuals appear willing to be treated by a PA instead of an MD under certain conditions. In this small study, willingness does not appear to be gender dependent and is not influenced by parenthood. Such work builds on other findings and suggests that the PA, as a construct, is a medical provider of interest across a growing number of western health systems.

## Competing interests

The authors declare that they do not have any competing interests.

## Authors’ contributions

LK is the principal investigator of the study, first authored the manuscript and performed the statistical analysis as a requirement of his PhD candidacy. RN, CPS and JHS contributed to the manuscript in terms of editorial consultancy. JHS also played the role of facilitator at the inclusion site to ensure our research assistants could undertake data collection. RSH served as the intellectual guidance of the willingness project. All authors read and approved the final manuscript.
